# Rod and Cone Pathway Signalling Is Altered in the P2X7 Receptor Knock Out Mouse

**DOI:** 10.1371/journal.pone.0029990

**Published:** 2012-01-10

**Authors:** Kirstan A. Vessey, Erica L. Fletcher

**Affiliations:** Department of Anatomy and Cell Biology, The University of Melbourne, Melbourne, Victoria, Australia; Oregon Health & Science University, United States of America

## Abstract

The P2X7 receptor (P2X7-R) is expressed in the retina and brain and has been implicated in neurodegenerative diseases. However, whether it is expressed by neurons and plays a role as a neurotransmitter receptor has been the subject of controversy. In this study, we first show that the novel vesicular transporter for ATP, VNUT, is expressed in the retina, verifying the presence of the molecular machinery for ATP to act as neurotransmitter at P2X7-Rs. Secondly we show the presence of P2X7-R mRNA and protein in the retina and cortex and absence of the full length variant 1 of the receptor in the P2X7-R knock out (P2X7-KO) mouse. The role of the P2X7-R in neuronal function of the retina was assessed by comparing the electroretinogram response of P2X7-KO with WT mice. The rod photoreceptor response was found to be similar, while both rod and cone pathway post-photoreceptor responses were significantly larger in P2X7-KO mice. This suggests that activation of P2X7-Rs modulates output of second order retinal neurons. In line with this finding, P2X7-Rs were found in the outer plexiform layer and on inner retinal cell classes, including horizontal, amacrine and ganglion cells. The receptor co-localized with conventional synapses in the IPL and was expressed on amacrine cells post-synaptic to rod bipolar ribbon synapses. In view of the changes in visual function in the P2X7-KO mouse and the immunocytochemical location of the receptor in the normal retina, it is likely the P2X7-R provides excitatory input to photoreceptor terminals or to inhibitory cells that shape both the rod and cone pathway response.

## Introduction

P2X receptors comprise a family of seven ligand-gated ion channels (P2X1-7) that are activated by extracellular ATP (eATP). They are non-selective and are permeable to Na^+^, K^+^ and Ca^++^, mediating excitatory neurotransmission throughout the central and peripheral nervous system [1], [2], [3]. The P2X7 receptor (P2X7-R) is a unique member of the P2X receptor family. It requires higher concentrations of eATP (10 mM) to become activated than other P2X receptors, and when stimulated by eATP it does conduct cations, but under special conditions it is permeable to larger molecules [4], [5] ultimately causing cell death. As a result activation of the P2X7-R, in particular on immune cells and glia, has been extensively studied as a mediator of inflammation, cell death and neural degeneration [6], [7], [8].

Very few studies have shown a specific role for the P2X7-R in mediating synaptic transmission on neurons. A functional role for the receptor has been implied from the excitatory actions of the relatively selective agonist, Bz-ATP, on neurons in the hippocampus [9], brainstem [10], and paraventricular nucleus [11]. However, there is little evidence directly confirming the involvement of P2X7-R in neurotransmission in these tissues using either specific antagonists and/or mice lacking the P2X7-R. Indeed, there is controversy over whether the receptor is expressed on neurons and involved in neurotransmission in the central nervous system at all [12], [13]. The aim of the current study was to determine if the P2X7-R has a neuronal function in another tissue of the central nervous system, the retina, using a P2X7-R knock out mice (P2X7-KO; [14], Pfizer).

The P2X7-R mRNA and protein is expressed by the mouse neural retina [15], [16], [17]. Further studies have shown a neurodegenerative role for the receptor on photoreceptors [18], retinal ganglion cells [19], [20] and also Müller glial cells [21], [22]. Only one study has provided some evidence for the involvement of the P2X7-R in normal (non degenerating) retinal synaptic transmission, based on the finding that Bz-ATP, a P2X receptor agonist with 10–30 fold selectivity for the P2X7-R, induces reversible changes in the electrophysiological response of the rat retina, in vivo [23]. Here we extend these findings by first showing that the retina expresses the novel vesicular transporter of ATP (VNUT) [24], which indicates that the molecular machinery required for ATP to act as a vesicle released, retinal neurotransmitter at P2X7-Rs is present in the retina. After determining the P2X7-R variant 1 is expressed in the retina of C57B6/J wildtype (WT) mice but not in P2X7-KO mice ([14]; Pfizer), we show that both the rod and cone post-photoreceptor pathway responses are enhanced in the P2X7-KO animal. The changes in the functional response observed in the P2X7-KO mouse are consistent with immunocytochemical evidence presented within the study, which suggest that the P2X7-R is present primarily on inner retinal neurons and that it is likely to have a role in modulating the inner retinal response.

## Methods

### Animals

All experiments involving animals were approved by the University of Melbourne animal experimentation ethics committee (Ethics ID number: 0911158). In addition, experiments adhered to the ARVO Statement for the Use of Animals in Ophthalmic and Vision Research. Animals were housed on a 12/12 hr light/dark cycle, cage luminance was <300 lux. All animals were aged between 60–90 days old. For *in vivo* experiments, mice were anaesthetised by intra-peritoneal administration of a mixture of ketamine (35 mg/kg) and xylazine (7 mg/kg). For *ex vivo* experiments, mice were first anaesthetised (as above) and euthanized by cervical dislocation.

C57B6/J wildtype (WT) mice were obtained from the University of Melbourne breeding colony. P2X7-KO mice (Pfizer, Groton, CT, USA)[14] were kindly donated by Prof. James Wiley (University of Sydney, NSW, Australia) and bred at the University of Melbourne. The P2X7-KO mice have an insertion in Exon13 of the P2X7-R gene which deletes from Cys506 to Pro532 in the C-terminus of the 595 amino acid protein. Thy1-HYFP mice, which have subpopulations of ganglion cells that express yellow fluorescent protein in cells, which produce the thy-1 promoter, were kindly donated by A/Prof Anthony Hannan (Howard Florey Institute, VIC, Australia) and bred at the University of Melbourne.

### Laser microdissection and mRNA analysis of the ATP vesicular transporter, VNUT

To determine whether the novel vesicular transporter for ATP, VNUT was expressed by neuronal classes in the retina, WT mouse retinae were fixed in 4% paraformaldehyde for 30 minutes, cryoprotected in graded sucrose, and stored in 30% sucrose in 0.1 M phosphate buffer (PB) at 4°C overnight. Retina were embedded in OCT (TissueTek), frozen and cut on a cryostat at 30 µm at −20°C and collected on Polylysine coated slides. Sections were washed and sequentially dehydrated in 50%–100% ethanol. Samples of either photoreceptor layer (PR) or inner nuclear/ganglion cell layer (INL/GCL) were laser microdissected and collected into 50 µL of lysis solution (buffer PKD) from the FFPE Qiagen kit for purification of total RNA from fixed tissue sections. Total RNA was extracted and primer specific RT-PCR was completed using the Qiagen One-Step RT-PCR kit. Primers to Rhodopsin and Thy1, were used to confirm the purity of the PR versus INL/GCL samples, respectively (Table 1). Primers specific to vesicular nucleotide transporter (VNUT) were designed (Table 1). The One step RT-PCR conditions were 50°C for 30 min, 95°C for 15 min, 95°C for 4 minutes (1 cycle each), 94°C for 30 sec, 55°C for 30 sec, 72°C for 1 min (46 cycles), 72°C for 10 minutes. Amplified products were separated by electrophoresis on a 1.5% agarose gel containing ethidium bromide and photographed under UV light (UVP BioDoc-It Systems).

**Table 1:** Oligonucleotide Primer List.

Gene	FW primer (5′-3′)	RV primer (5′-3′)	Size(bp)	Comment
Rhodopsin	AGC AGC AGG AGT CAG CCA CC	CCG AAG TTG GAG CCC TGG TG	145	Photoreceptor specific gene
Thy1	AAT GAT GGG GAA AGG GGT AG	GGA GAG GAT CCT TGG GAA AG	377	Inner retina specific gene
VNUT	AAA GAC CTT GTC CTG GCC CT	TTG AAG ACC CAG CCC TTG GA	212	Unknown retinal location
P2X7-R (var1-3)	ATA ATA TCC ACT TCC CCG GC	GTC CGC TTT TCC ACA TTG TT	343	Central region of P2X7-R common to splice var 1, 2 and 3
P2X7-R (var1) Primer pair A	CAT CAC CAC CTC CAA GCT CT	TAT ACT GCC CCT CGG TCT TG	248	C-term region of P2X7-R var 1 From start of deletion in KO to end
P2X7-R (var1) Primer pair B	CAC CGT GCT TAC AGG TGC TA	TAT ACT GCC CCT CGG TCT TG	127	C-term region of P2X7-R var 1 After deletion in KO to end

### Standard RT-PCR for P2X7-R mRNA splice variants in WT and P2X7-KO tissues

To confirm that the P2X7-R mRNA is expressed in WT and not P2X7-KO retina we used standard RT-PCR. Four retinae or one cortex, per genotype, was used for RNA extraction using an RNeasy Mini Kit (Qiagen, Germany). Genomic DNA was digested using deoxyribonuclease I (Sigma-Aldrich kit, AMP-D1) and the RNA repurified. Reverse Transcription, using random hexamer primers, was used to generate copy DNA (Superscript III First-Strand Synthesis system, Invitrogen, Australia). A control for genomic DNA contamination was prepared at the same time by completing the reverse transcription on each mRNA sample in the absence of the Superscript III enzyme. Standard PCR using a TAQ DNA polymerase kit (Scientifix, Australia) was used to amplify various regions of the P2X7-R to assess receptor expression in the retina and cortex of mice.

Four splice variants of P2X7-R mRNA have been found in the mouse. Only variant 1 encodes the full length 595 amino acid protein, while variant 2, 3 and 4 are truncated versions that end at or before Exon 12. As a result receptor protein produced from variant 2, 3 and 4 mRNA would lack the carboxyl tail of the P2X7-R and would likely have impaired cation channel function [25]. A previous study has shown that there may be read through of C-terminal P2X7-R variant 1 mRNA after the insertion/deletion in the P2X7-KO ([14], Pfizer) mouse such that a P2X7-R with slightly altered electrophysiological properties is generated [26]. To assess whether P2X7-R splice variants other than variant 1 are expressed in the WT and P2X7-KO mouse retina and also whether read through of variant 1 C-terminal mRNA occurs in the P2X7-KO mouse retina, primers were designed against: 1) the central region of the P2X7-R which is common to variants 1, 2 and 3; 2) the C-terminus from within the start of the disruption in the P2X7-KO to the end of the mRNA (variant 1 only, primer pair A); and 3) the C-terminus from after the insertion deletion in the P2X7-KO mouse to the end of the receptor (variant 1 only, primer pair B). For all primer pairs, PCR conditions were 95°C for 4 minutes (1 cycle), 95°C for 30 sec, 55°C for 30 sec, 72°C for 1 min (30 cycles), 72°C for 2 minutes. Amplified products were separated by electrophoresis and photographed under UV light.

### Western Blotting for P2X7-R in WT and P2X7-KO tissues

To determine the expression profile of P2X7-R splice variants in the WT and P2X7-KO mouse and confirm the specificity of the antibody used, a Western blot using mouse retinae and cortex was completed as previously described [27]. An antibody specific for the extracellular, N-terminal region of the P2X7-R peptide (Alomone labs, Cat. no#APR008) was used as it should bind all four splice variants of the P2X7-R. Retinae and cortex from both P2X7-KO and WT mice were dissected and homogenised in a HEPEs buffer containing 40 mM HEPEs, 320 mM sucrose, pH 7.5, and a protease inhibitor cocktail. Samples were centrifuged (7,500 g for 1 min), then the supernatant was collected and re-centrifuged (13,000 g for 15 min) and subsequent the pellet resuspended in HEPEs buffer. Samples were diluted 1 part sample to at least 2 parts Tris-SDS buffer (Tris 0.5 M pH 6.8, glycerol, 10% SDS, 0.5% bromophenol blue, 0.5% mercapto-ethanol), so that 70 µg of protein per sample could be loaded onto the gel. Samples were boiled for 4 minutes and loaded onto an Acrylamide/Bis gel (12%), along with a molecular weight marker (Odyssey®-Licor; Cat no# 928-4000). Protein was separated on the gel by electrophoresis and then transferred to a nitrocellulose membrane (Bio-Rad Laboratories, USA). The membrane was placed in a SNAP i.d. system (Millipore), blocked in Tris-buffered saline with Tween 0.05% (TBST) containing 0.5% skim milk powder, then incubated for 10 min in primary antibodies (Rabbit anti-P2X7-receptor (extracellular); dilution 1∶150; Alomone labs, Cat. no#APR008: Mouse anti-GAPDH; dilution 1∶3000; Sigma-Aldrich, Cat no#G8795). The membrane was washed in TBST, incubated for 10 min in secondary antibody (Goat anti-rabbit IgG-680CW; 1∶3000; Odyssey-Licor-Millenium; Cat no#926-68021: Goat anti-mouse IgG-800CW; 1∶3000; Odyssey-Licor-Millenium; Cat no#926-32210) and washed again in TBST. The membrane was imaged using an OdysseyClx Infrared imaging system (Licor biosystems). .

### Visual function of WT and P2X7-KO mice

#### Electroretinogram

To assess the functional responses of retinal neurons, electroretinograms (ERGs) were recorded, *in vivo*, under dark adapted conditions from P2X7-KO (n = 12) and WT (n = 9) mice as previously described [28]. The ERG was recorded using a Ag/AgCl electrode placed on the cornea and a reference electrode placed in the mouth. Responses were amplified (gain×5000; −3 dB at 1 Hz and 1 kHz, ADInstruments, Castle Hill, New South Wales, Australia) and digitized at 10 kHz. Full field ERG, mixed rod and cone responses were elicited using a commercial photographic flash unit (Mecablitz 60CT4, Zirndorf, Germany) delivered through a Ganzfeld bowl and attenuated by neutral density filters so that flash intensity could be modified from −4.2 to 2.1 log cd.s/m^2^. At the highest three intensities (1.5 to 2.1 log cd.s/m^2^) two consecutive flashes (0.8 sec inter stimulus interval) were used to assess the rod and cone responses independently [29], [30].

The amplitude of the ERG a-wave and also b-wave from the mixed rod and cone response was plotted as a function of stimulus intensity. The response was modelled using a sigmoidal curve function [31], [32] and fit by minimisation of least squares. The equation used was:
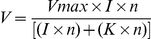
where, V is the response in voltage (µV) and I is the stimulus intensity (log cd.s/m^2^). V_max_ is the maximum response amplitude (µV), K is the intensity of the stimulus at half V_max_ (log cd.s/m^2^) and n, which was constrained to unity, is related to the slope of the function.

The independent rod and cone ERGs obtained using the twin flash protocol at the higher intensities (1.5 to 2.1 log cd.s/m^2^) were analysed using equations described in detail in Weymouth and Vingris, 2008 [33]. In brief, the rod a-wave was fitted using the PIII model modified from Hood and Birch (1990) to assess the Rmax (µV), the saturated amplitude of the PIII, S (sensitivity), the gain of the phototransduction process (m^2^·cd^−1^·s^−3^) and td (seconds), the recording latency following stimulation. The post photoreceptor response of the rod ERG, the b-wave, was isolated by subtracting the rod PIII from the raw rod waveform and fitted (PII model) using an inverted gamma function to assess the amplitude of the response, rood PII Rmax (µV) and the time until the maximum response was reached, implicit time (ms). The cone post photoreceptor response (cone PII) was fitted using an inverted gamma function to assess amplitude of the response, cone PII Rmax (µV); and the time until the maximum response was reached, implicit time (ms).

#### *In vivo*, visual spatial threshold measurements

The visual responses of adult WT and P2X7-KO mice were assessed *in vivo* by monitoring head tracking of sinusoidally modulated gratings in a virtual rotating optokinetic drum (Optomotry, CerebralMechanics, Lethbridge, Alberta, Canada) [34]. Spatial frequency thresholds were determined using a simple staircase protocol that varied the spatial frequency of gratings rotating at a constant velocity (12 deg s^−1^). The average spatial frequency cutoff (cycles/degree) was defined as the highest spatial frequency that elicited a reliable head tracking response to a 100% contrast grating. Mice were tested at a photopic mean luminance (100 cd m^−2^). All measurements were made by an observer blind to stimulus condition.

### Statistical analysis

Data manipulation and curve fitting was completed using Excel (Microsoft Office Professional Edition 2003). Graphing and statistical analysis was performed using GraphPad Prism 4 (GraphPad Software, San Diego, CA, USA). ERG and visual acuity data from WT and P2X7-KO mice are presented as the mean ± the standard error of the mean (SEM). Data were compared using a Student's t-test performed using GraphPad Prism and differences between the mean responses were considered significant if p<0.05.

### Gross Histology of WT and P2X7-KO retina

The morphology of WT and P2X7-KO retinas was ascertained using paraffin sections stained with hematoxylin and eosin. Whole eyes were fixed overnight in 4% paraformaldehyde containing 3% sucrose, 5% acetic acid and 60% ethanol. Eyes were then dehydrated in graded alcohols before being embedded in paraffin wax. Retinas were then sectioned at 4 µm, placed on polylysine® coated slides and incubated overnight at 37°C. Sections were deparaffinized, stained with Mayer's hematoxylin and eosin and coverslipped. An Axioplan microscope (Zeiss, Göttingen, Germany) was used to view retinal sections and images were captured by using a digital camera and computer software (SPOT, version 3.5.2, Diagnostic Instruments, Perth, WA, Australia). Images were converted to grayscale and adjusted for white levels, brightness, and contrast with Adobe Photoshop CS4 (Adobe Systems, San Jose, CA).

### Histological analysis of P2X7-R expression in the WT retina

#### Immunohistochemistry

Fluorescence immunohistochemistry was used to co-localise the P2X7-R with cell markers in the retina using previously described techniques [28]. The posterior eye cups of WT mice were fixed and processed as for laser microdissection above. Tissues were sectioned transversely at 14 µm on a cryostat and collected on polysine® coated slides and stored at −20°C. For labelling, slides were defrosted and washed in PB. Sections were coated in a blocking solution (10% Normal goat serum (NGS), 1% bovine serum albumin (BSA), 0.5% Triton-X in PB) for one hour before incubation overnight, at room temperature, in primary antibody (Table 2) dissolved in antibody buffer (3% NGS, 1% BSA, 0.5% Triton-X in PB). Slides were washed in PB and incubated in the dark for one hour in secondary antibody (Table 2). The sections were washed in PB, mounted in Mowiol and covered with a glass coverslip.

**Table 2:** Antibody List.

Primary Antibody	Dilution	Company
Rabbit anti-P2X7-receptor (extracellular)	1∶500	Alomone Labs.; Cat. No# APR 008
Rabbit anti-P2X7-receptor (C-terminus)	1∶1000	Alomone Labs.; Cat. No# APR 004 & Sigma; Cat No# P8232
Mouse anti-Calbindin D28k	1∶4000	Schwant; Cat. No# 300
Mouse anti-postsynaptic density 95 (PSD-95, clone 7E3-1B8)	1∶5000	Affinity Bioreagents; Cat. No#MA1-046
Guinea pig anti-GABA	1∶500	Millipore/Chemicon.; Cat. No# AB175
Mouse anti-Bassoon (clone SAP7F407)	1∶100	Assay Designs; Cat.No#ADI-VAM-PS003
Mouse anti-Protein Kinase C	1∶400	Sigma; Cat. No# P 5704
Mouse anti-Calretinin 6B3	1∶1000	Schwant; Cat. No# 63B
Mouse anti-C-Terminal Binding Protein-2 (CtBP2; Ribeye)	1∶2000	BD Transduction Labs; Cat. No# 612044
Mouse anti-ZNP-1	1∶2000	ZIRC; Cat. No# ZDB-ATB-081002-25
Mouse anti-Green Fluorescent Protein	1∶5000	Invitrogen, Cat. No#A11120
Mouse anti-Glutamine Synthetase (GS, clone GS-6)	1∶1000	Millipore/Chemicon.; Cat. No# MAB302
Mouse anti- glial fibrilliary acidic protein (GFAP, clone GF 12–24)	1∶5000	Millipore/Chemicon.; Cat. No# CBL411

Secondary Antibody	Dilution	Company
Goat anti-rabbit IgG Alexa 488,594,647	1∶500	Molecular probes, Invitrogen
Goat anti-mouse IgG Alexa 488,594	1∶500	Molecular probes, Invitrogen
Goat anti-guinea pig IgG Alexa 594	1∶500	Molecular probes, Invitrogen

Retinae were viewed and imaged using a Zeiss LSM-5 confocal microscope (Zeiss, Germany). Oil objectives (40X and 63X) were used and fluorophore labelled sections were captured at a resolution of 1024 by 1024 pixels using Ziess LSM image browser software and an appropriate fluorescence filter (Alexa TM 594/CY3: excitation 568 nm, emission filter 605/32; Alexa TM 488/FITC: excitation 488 nm, emission filter 522/32). Red and green fluorescence was scanned separately and adjusted for black levels and contrast with Adobe Photoshop.

Co-localisation analysis was completed using Image J 1.43 Freeware (NIH, USA). For co-localisation of GABA positive cell bodies with P2X7-R-immunoreactivity (P2X7-R-IR), the Image J plug-in, RG2B colocalisation was used and the number of co-localised cells were counted and expressed as a percentage of the total number of GABA-IR cells. For the observed co-localisation of P2X7-R-IR puncta with ribeye or bassoon positive puncta in the IPL, the Image J plug-in, RG2B colocalisation was used and the number of co-localised puncta were counted using the image J macro ITCN. The number of co-localised puncta were then expressed as a percentage of the total number of ribeye or bassoon positive puncta in specifically the IPL. For comparison, a random co-localisation analysis was performed on images in which the P2X7-R-IR image was flipped horizontally with regard to the ribeye or bassoon image. The same approach for identifying observed and randrom co-localisation was applied when quantifying the number of discrete P2X7-R-IR puncta co-localised with astrocytes or Müller cells/processes, however in these cases the number of co-localised P2X7-R-IR puncta were expressed as a discrete number per imaged area (mm^2^) that was assessed, ie. number of colocalised puncta/retinal area mm^2^.

#### Electron microscopy

Pre-embedding immunocytochemistry for electron microscopy was completed to determine the ultrastructural cellular location of the P2X7-receptor in the IPL. The method has been described previously [35]. Vibratome sections of WT mouse retina were blocked then incubated in the rabbit anti-P2X7 antibody (1∶500) for 4 days at 4°C. A biotinylated goat–anti-rabbit secondary antibody (1∶100) was applied for 2 h. To visualise the secondary antibody, a Vectastain Elite ABC Kit (Vector Laboratories, Burlingame, CA, USA) and 3,3′-diaminobenzidine (DAB) reaction (Metal Enhanced DAB Substrate Kit, Pierce, USA) was used. Sections were post-fixed with 2.5% (v/v) glutaraldehyde in cacodylate buffer for 2 h at 4°C. The DAB reaction product was silver intensified and gold toned and sections were then incubated in 0.5% OsO_4_. Sections were dehydrated and embedded in Epon resin. Ultrathin sections (70 nm) were collected on Formvar-coated copper grids, contrasted with uranyl acetate and lead citrate solutions, and viewed with a Phillips CM120 electron microscope.

## Results

### The mouse retina has the molecular machinery for vesicular transmission of ATP

**Figure 1:**
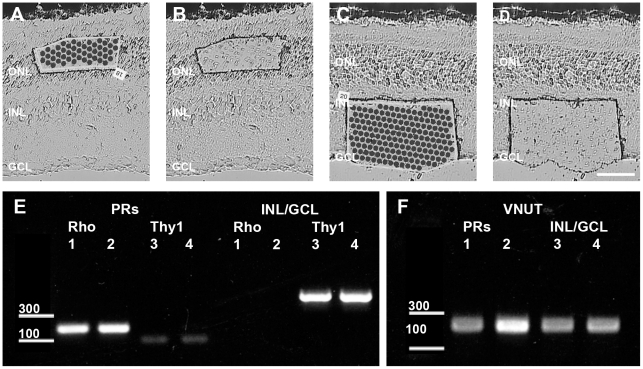
PCR for the vesicular transporter for ATP, VNUT mRNA expression using laser microdissected retinal samples. (A–B) The outer nuclear layer (ONL) region targeted (A) and collected (B) for capture of photoreceptor mRNA. (C–D) The inner retinal region targeted (C) and collected (D) for capture of inner nuclear layer (INL) and ganglion cell layer (GCL) mRNA. E) RT-PCR was completed on laser microdissected samples from two WT retinas for both photoreceptor (PRs; 1&2) and INL/GCL (3&4) samples. Rhodopsin mRNA was present in photoreceptor samples and not INL/GCL sample, while Thy1 mRNA was present in INL/GCL samples and not the photoreceptor sample, confirming sample purity. (F) The mRNA for the vesicular transporter for ATP, VNUT was present in the retina in both the photoreceptor and the INL/GCL samples.

Traditional neurotransmission involves the production of molecular machinery for vesicular storage, release and mechanisms for degradation of the chemical mediator. A vesicular mechanism of ATP release in the retina has not been shown and multiple mechanisms of “non-traditional” ATP release have been proposed, including hemi-channel release of ATP from the retinal pigment epithelium (RPE) [36] and mechanical stimulation induced ATP release from glia [37]. Recently, the vesicular transporter for ATP, Vesicular Nucleotide Transporter (VNUT) was characterised [24]. To determine if VNUT was expressed in the mouse retina, laser microdissection was used to collect photoreceptor (PR; Figure 1A–B) and inner nuclear/ganglion cell layer (INL/GCL; Figure 1C–D). Sample separation of PR and INL/CGL was confirmed by RT-PCR for rhodopsin, expressed exclusively by PRs, and Thy-1, expressed by cells in the INL/GCL (Figure 1E). RT-PCR for VNUT from the PR and INL/GCL samples showed that the vesicular transporter for ATP is produced by cells in the inner and outer retina (Figure 1F). This indicates that ATP is likely to be released in a vesicular (VNUT) dependent mechanism from photoreceptors and inner retinal cells, and thus is able to act as a traditional neurotransmitter at P2X-receptors including the P2X7-R.

### P2X7-R variant 1 mRNA and protein is present in WT but absent in P2X7-KO mouse retina

**Figure 2:**
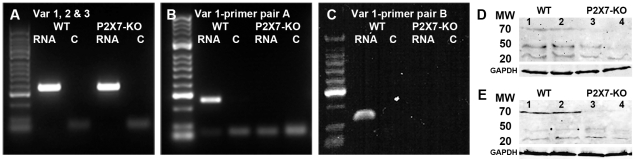
PCR and western blot analysis of P2X7-R splice variants in WT and P2X7-KO retina. To assess whether P2X7-R splice variants other than the full length, variant 1 are expressed in the WT and P2X7-KO mouse retina and also whether read through of variant 1 C-terminal mRNA occurs in the P2X7-KO mouse retina, PCR for (A) the central region of the P2X7-R which is common to variants 1, 2 and 3; (B) the C-terminus from within the start of the disruption in the P2X7-KO to the end of the mRNA (variant 1 only, primer pair A); and (C) the C-terminus from after the insertion deletion in the P2X7-KO mouse to the end of the receptor (variant 1 only, primer pair B) was completed. P2X7-R variant 1 C-terminal mRNA was absent (B–C) in the P2X7-KO mouse but N-terminal mRNA variants (likely variant 2 or 3) were still expressed (A). MW, molecular weight marker and the bright bar is 300 bp. (D–E) Protein analysis of P2X7-R splice variant expression was completed using Western blots of mouse retina (D) and cortex (E). An N-terminal specific P2X7-R antibody detected the protein transcript of variant 1 mRNA in WT samples 1 and 2 but not P2X7-KO samples 3 and 4 (68.41 kDa) of both retina and cortex. Protein for variant 2/3 (50.69 kDa/49.38 kDa) and 4 (17.2 kDa) was expressed in both WT and P2X7-KO retina and cortex. A monoclonal antibody against GAPDH was applied to the same blots and indicates that similar amounts of protein were loaded for WT and P2X7-KO samples.

In order to determine the role of the P2X7-R in the retina using the P2X7-KO mouse ([14]; Pfizer), it was first important to determine the absence of the receptor in the knock out mouse (Figure 2A–D). There are four splice variants of P2X7-R mRNA that have been found in the mouse and only variant 1 encodes the full length 595 amino acid protein while variant 2, 3 and 4 are truncated versions that end before the insertion/deletion in the P2X7-KO mouse ([14]; Pfizer). RT-PCR analysis of WT and P2X7-KO retina indicated that P2X7-R variant 1 mRNA is usually expressed in the WT mouse retina. In contrast, P2X7-R variant 1 mRNA is not expressed in the retina of the knock out animal (Figure 2B–C). A previous study has shown that there may be read through of C-terminal P2X7-R variant 1 mRNA after the insertion/deletion in the P2X7-KO ([14], Pfizer) mouse such that a P2X7-R with slightly altered electrophysiological properties is generated in the hippocampus [26]. Our data indicate that in the P2X7-KO retina there is no read through of the P2X7-R variant 1 gene generating C-terminal mRNA, either within (Figure 2B) or after the insertion/deletion (Figure 2C). However, using primers designed against the central region of the P2X7-R mRNA, which would amplify mRNA from variants 1 and/or 2 and/or 3, a product was detected in both the WT and the P2X7-KO retina (Figure 2A). This indicates that variant 2 and/or 3, but not variant 1, P2X7-R mRNA is expressed in the P2X7-KO animal. [25]

The protein expression of the P2X7-R splice variants was then assessed, using a polyclonal antibody generated against the extracellular, N-terminal region of the P2X7-R peptide, by western blot of retinae (Figure 2D) and cortex (Figure 2E) from WT and P2X7-KO animals. The P2X7-R antibody should detect P2X7-R protein from variant 1, 2, 3 and 4 mRNA. The antibody labelled protein of around 70 kDa, corresponding to P2X7-R variant 1 peptide, in protein from WT tissue (samples 1 & 2) but not P2X7-KO tissue (samples 3 & 4). In addition, the antibody detected protein of around 50 kDa corresponding to variant 2 and/or 3, and 20 kDda protein corresponding to variant 4 of the P2X7-R in both the retinae and cortex of WT and P2X7-KO mice. A monoclonal antibody against GAPDH was also used on the same blots as a protein loading control and indicates similar amounts of protein from WT and P2X7-KO retina and cortex samples were loaded. Quantification of the density of the P2X7-R bands, relative to the GAPDH control, indicated that while the 70 kDa band corresponding to P2X7-R variant 1 was absent in retinae and cortex of P2X7-KO mice, there was no change in the expression of the other splice variants between WT and P2X7-KO mice (n = 3 samples tested per genotype, data not shown). As receptor protein produced from variant 2, 3 and 4 mRNA would lack the carboxyl tail of the P2X7-R, the flow of ions and general function of the P2X7-R would be severely compromised in the P2X7-KO mouse [25], [38]. Therefore, any changes in retinal function in the P2X7-KO mouse would be due to absence of specifically variant 1 of the P2X7-R.

### Neuronal function is altered in the P2X7-KO mouse

**Figure 3:**
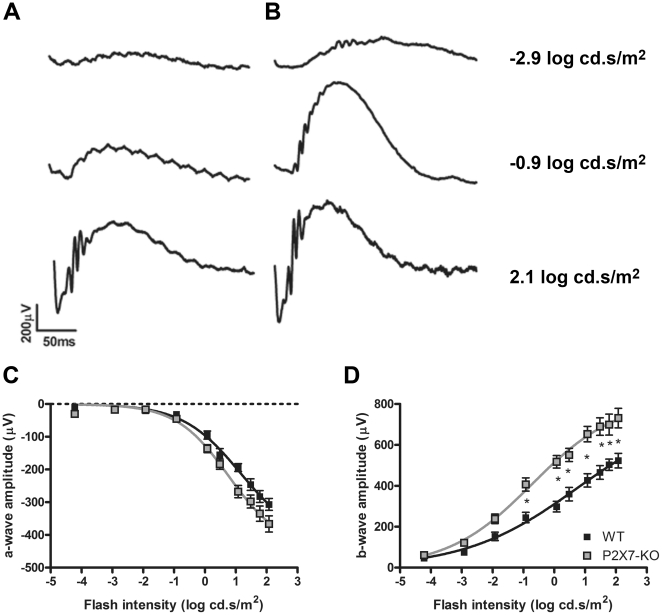
The mixed rod and cone ERG is altered across a range of light intensities in the P2X7-KO mouse. Mice were dark adapted overnight and mixed (rod and cone) ERG responses were recorded in response to full field flashes from intensity −4.2 to 2.1 log cd.s/m^2^. (A–B) Representative mixed ERG waveforms for (A) WT and (B) P2X7-KO mice are presented. (C) There was no change in the amplitude of the rod photoreceptor derived component, a-wave, as a function of intensity. (D) The amplitude of the post-photoreceptor response, the b-wave was larger in P2X7-KO mice than in WT mice across almost all intensities tested. * indicates p<0.05, a significant difference between WT and P2X7-KO.

The ERG was used to determine whether changes in neuronal function occurred in the P2X7-KO mouse. Mice were dark adapted overnight and mixed (rod and cone) ERG responses were recorded in response to full field flashes from intensity −4.2 to 2.1 log cd.s/m^2^. Representative mixed ERG waveforms for WT and P2X7-KO mice are presented (Figure 3 A and B respectively). The average responses from the mixed waveforms are presented in Figure 3 C–D, and analysis indicates that there was no change in the amplitude of the rod photoreceptor derived component of the ERG, the a-wave as a function of intensity (Figure 3C; −4.2 to 2.1 log cd.s/m^2^; Two Way ANOVA for stimulus intensity p<0.0001 and genotype p = 0.14). However, the amplitude of the post-photoreceptor neural response, the b-wave, was larger in P2X7-KO mice than in WT mice across almost all intensities tested (Figure 3D; −4.2 to 2.1 log cd.s/m^2^; Two Way ANOVA for stimulus intensity p<0.0001 and genotype p<0.0001; post-test between WT and P2X7-KO, * p<0.05 for intensities greater than −0.9 log cd.s/m^2^).

**Figure 4:**
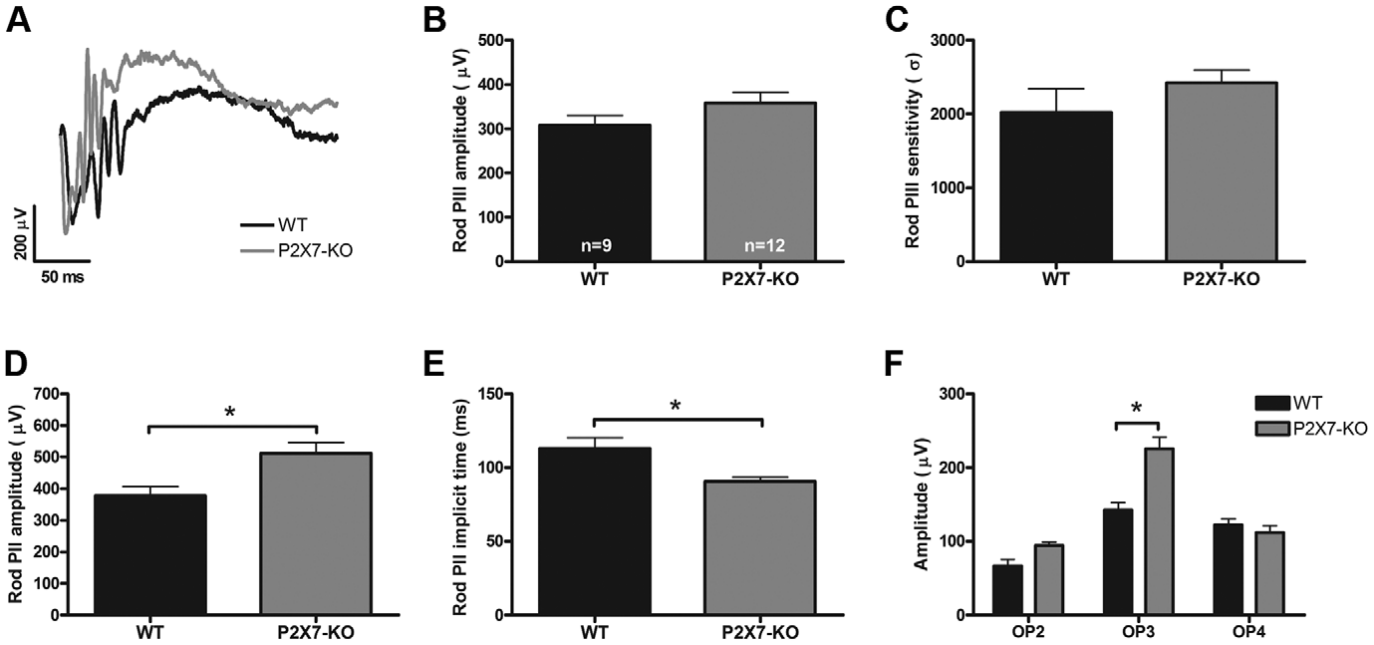
The function of the rod pathway is altered in the P2X7-KO mouse. (A) Representative waveforms of the rod ERG recorded from WT (black) and P2X7-KO mice (grey) in response to a 2.1 log cd.s/m^2^ intensity flash. The photoreceptor derived, rod PIII (modelled a-wave) was not altered in either (B) amplitude or (C) sensitivity in the P2X7-KO mouse when compared with the WT response. The post-photoreceptor, rod PII (modelled b-wave) was found to be (D) larger in amplitude and (E) faster to reach a maximum response. (F) The amplitude of the third of the inner retinal derived oscillatory potentials was significantly enhanced in the P2X7-KO mouse. * indicates p<0.05, a significant difference between WT and P2X7-KO.

At the highest flash intensities (1.5 to 2.1 log cd.s/m^2^), the rod and the cone pathway responses were isolated. Representative rod ERG waveforms for WT and P2X7-KO mice are presented in Figure 4A. There was no change in the rod photoreceptor response (a-wave, PIII), which was analysed for alterations in amplitude (Figure 4B) and sensitivity (Figure 4C). However, the rod post-photoreceptor response (b-wave, PII) was significantly larger (Figure 4D) and faster (Figure 4E) in the P2X7-KO mouse. This indicates that in the P2X7-KO mouse, rod bipolar cells undergo larger depolarization in response to light than in the WT mouse. Further analysis of the post-photoreceptor response, the oscillatory potentials (OPs), revealed that although there was no change in the timing of the OPs (data not shown) there was a significant increase in the amplitude of the OPs, specifically OP3 (Figure 4F). The change in OP3 is likely to be driven primarily by an alteration in oscillations of the membrane potential of amacrine cells in the P2X7-KO mouse.

**Figure 5:**
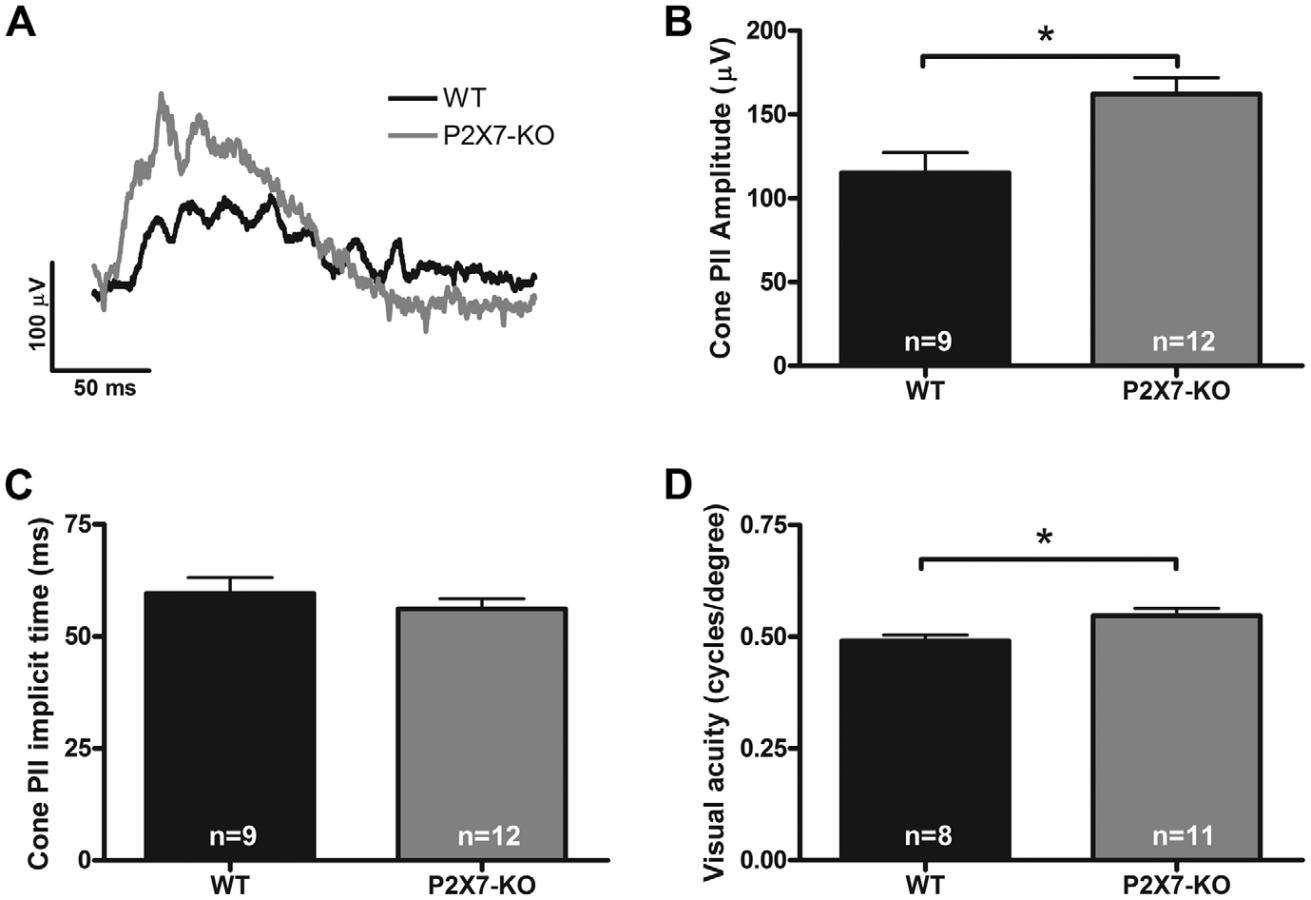
The function of the cone pathway is altered in the P2X7-KO mouse. (A) Representative waveforms of the cone ERG recorded from WT (black) and P2X7-KO mice (grey) in response to a 2.1 log cd.s/m^2^ intensity flash. The post-photoreceptor, cone PII (modelled b-wave) was found to be larger in (B) amplitude but not faster to reach a maximum response (C) in the P2X7-KO mouse when compared with the WT response. (D) The photopic visual acuity, assessed using the optokinetic reflex to determine the spatial frequency threshold, was higher in the P2X7-KO mouse than in the WT. * indicates p<0.05, a significant difference between WT and P2X7-KO.

The cone pathway was also assessed in WT and P2X7-KO mice and representative cone ERG waveforms are presented in Figure 5A. Due to the paucity of cones photoreceptors in the mouse retina the cone derived a-wave could not be measured. However, the cone post-photoreceptor response (b-wave, PII) was found to be enhanced in amplitude (Figure 5B) without a change in timing (Figure 5C) in the P2X7-KO mouse. In addition, visual behavioural testing of P2X7-KO mice under photopic conditions showed that they had a small but significant increase in spatial frequency threshold compared to age matched WT mice (Figure 5D). These data imply that in the normal retina the P2X7-R (variant 1) plays a role in neuronal transmission within the rod and cone pathways.

**Figure 6:**
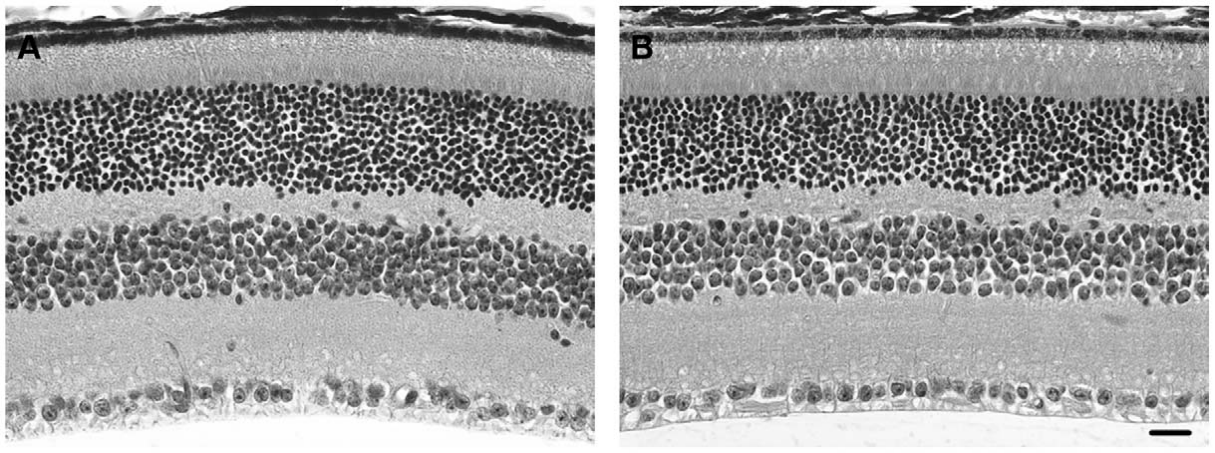
The gross morphology of the adult WT and P2X7-KO central retina is similar. Transverse sections of paraffin embedded retina from (A) WT and (B) P2X7-KO mice were stained with hematoxylin and eosin for comparison. The gross retinal morphology was similar in both mice. Scale bar, 20 microns.

The gross retinal morphology of the P2X7-KO mouse was also investigated to ascertain whether absence of the P2X7-R affected retinal structure (Figure 6). Transverse sections of paraffin embedded, central retina from WT (Figure 6A) and P2X7-KO (Figure 6B) mice were stained with hematoxylin and eosin and compared for layer thickness and general alterations in structure. The gross retinal morphology was qualitatively similar in both mice, suggesting that the changes in retinal function observed in the P2X7-KO mouse are unlikely to be due to overt changes in retinal structure.

### The P2X7-R is located on neurons of the WT mouse retina

**Figure 7:**
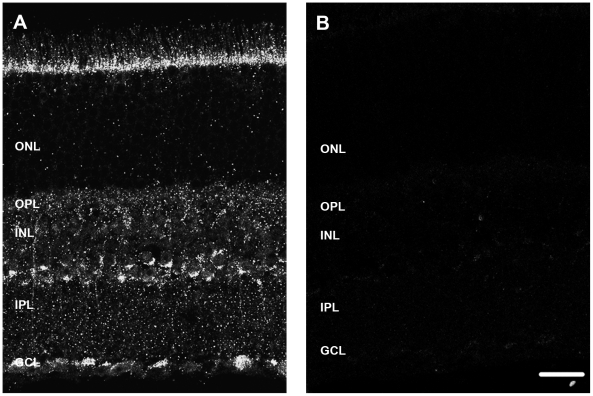
P2X7-R-immunoreactivity in the mouse retina assessed using an N-terminal specific antibody. (A) Immunohistochemistry on a transverse retinal section from a WT mouse showing P2X7-R immunoreactivity in the outer plexiform layer (OPL), inner nuclear layer (INL), and ganglion cell layer (GCL). (C) The retina does not label with the P2X7-R antibody following incubation with P2X7-R specific peptide. Scale bar, 20 microns.

Fluorescence immunohistochemistry was used to detect P2X7-R immunoreactivity (P2X7-R-IR) in the retina. Antibodies generated against the amino acid residues 576–595 from the C-terminal region of the rat P2X7-R and therefore against specifically Variant 1 were tested (Sigma-Aldrich, Cat. no# P8232 and Alomone, Cat. no# APR004). However, they labelled similarly in WT and P2X7-KO retina (data not shown) as has been shown previously in other tissues [12], even though the P2X7-KO mouse lacks variant 1 of the receptor. Further studies with these antibodies were not undertaken. The extracellular, N-terminal P2X7-R antibody directed against amino acid residues 136–152 of the receptor (Alomone labs, Cat. no#APR008) that was used for western blotting was also found to label similarly in both WT (Figure 7A) and P2X7-KO mice (data not shown). This was not unexpected given that although the P2X7-R variant 1 is no longer present in the P2X7-KO mouse, the remaining P2X7-R splice protein variants are still present. As the extracellular, N-terminal antibody labelled proteins of the correct size in the WT and P2X7-KO in the western blot (Figure 2D), it was used to localise P2X7-R expression in WT retina using fluorescence immunohistochemistry.

**Figure 8:**
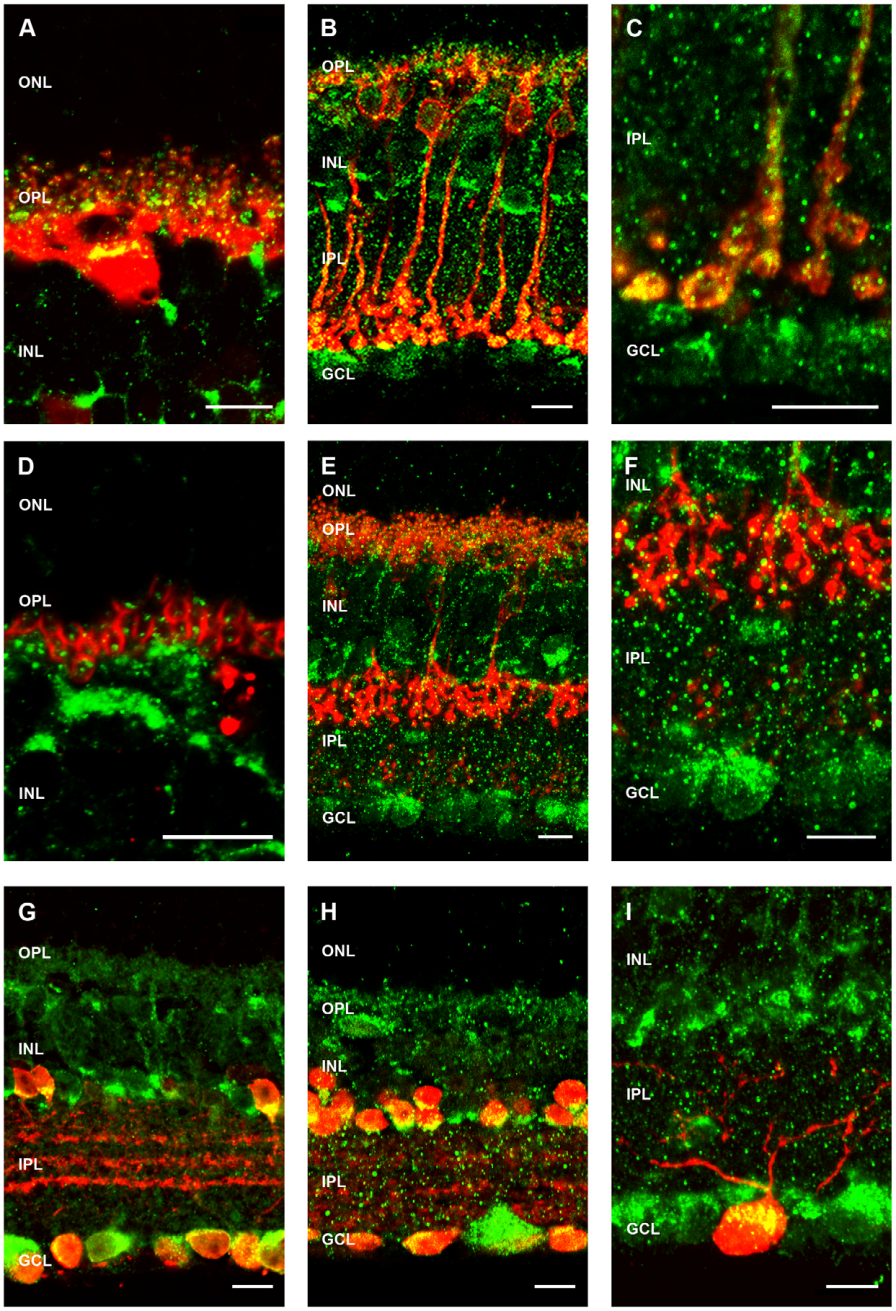
Immunohistochemistry for P2X7-R-immunoreactivity (IR) in the mouse transverse retina. (A) P2X7-R IR (green) colocalises with horizontal cells labelled with Calbindin (red) and their terminals. (B) P2X7-R IR (green) colocalises with rod bipolar cells labelled with PKC (red). (C) Inset of rod bipolar cell terminals. (D) P2X7-R IR (green) puncta are discretely expressed within photoreceptor terminals labelled with PSD-95 (red). (E) Cone bipolar cells (Type 2 and 6) labelled with a ZNP-1 antibody (green) colocalise with P2X7-R IR (red) including both the cone bipolar cell dendrites and terminals (F). P2X7-R IR (green) colocalises with amacrine cells labelled with calretinin (G) and in particular GABAergic amacrine cells (H). (I) Ganglion cells labelled with an antibody against GFP in the Thy1-HYFP mouse (pseudo-coloured red) were P2X7-R IR (pseudo-coloured green). Scale bar, 10 microns.

P2X7-R-IR was found on many cell types in the WT retina in the INL and GCL and also as discrete puncta within the OPL and IPL, as well as on the outer segments of the photoreceptors (Figure 7A). This labelling could be blocked by pre-absorption with the P2X7-R peptide sequence used to generate the antibody (Figure 7B). Co-labelling with cell markers specific for distinct neuronal classes in the WT retina showed that P2X7-R-IR (green) co-localised with many neurons within the inner retina (Figure 8). P2X7-R-IR (green) colocalised with horizontal cells labelled for Calbindin (red) and was expressed as discrete puncta in the OPL associated with horizontal cell dendrites/terminals (Figure 8A). Consistent with this, P2X7-R-IR (green) puncta were found within photoreceptor terminals labelled with PSD-95 (red; Figure 8D) in the OPL. In the inner retina, rod bipolar cells (Figure 8B) and in particular their terminals labelled for PKC (red; Figure 8C). Similarly Type 2 and 6 cone bipolar cells labelled for ZNP [39] (red; Figure 8E) and their terminals (Figure 8F) co-localised with P2X7-R-IR (green). In addition, amacrine cell populations immunoreactive for the cell markers Calretinin (red; Figure 8G) and the neurotransmitter GABA (red; Figure 8H) expressed P2X7-IR. The number of GABA-IR amacrine cells that expressed P2X7-R-IR were quantified and while nearly all GABA positive displaced amacrine cells in the ganglion cell layer expressed P2X7-R-IR (95%±3.1%), only 80% of GABA-IR cells in the inner nuclear layer expressed P2X7-R-IR (80.6%±6.9%). This suggests that only sub-populations of amacrine cells express the P2X7-R. Finally, ganglion cells, from mice that express HYFP driven in ganglion cells by the Thy-1 promoter [40] and labelled with an antibody against GFP (pseudo-coloured red), were found to express P2X7-R-IR (pseudo-coloured green; Figure 8I).

### The P2X7-R is expressed by amacrine cells post-synaptic to bipolar cell terminals

**Figure 9:**
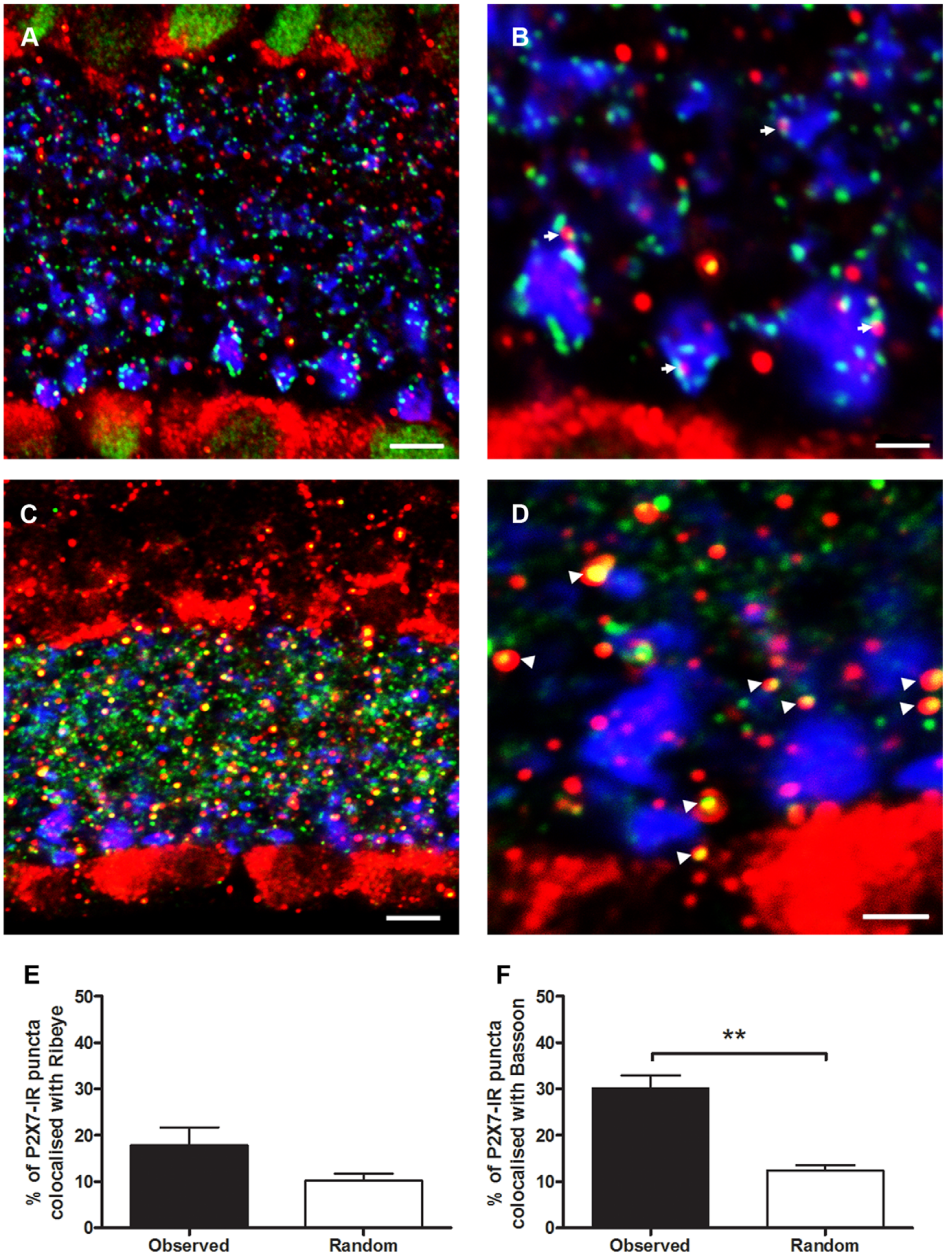
Immunohistochemistry for P2X7-R-immunoreactivity (IR) in the WT mouse transverse retina is associated with ribbon synapses and colocalises with coventional synapses in the IPL. (A) P2X7-R IR (red) is associated with but does not colocalise with ribbon synapses (ribeye, green) on bipolar cell terminals (VGLUT, blue). (B) Magnified inset from A, arrows indicate ribbon synapses associated with P2X7-R IR puncta. (C) P2X7-R IR (red) colocalises with conventional synapses (bassoon, green) distinct from bipolar cell terminals (VGLUT, blue). (D) Magnified inset from C, arrows indicate conventional synapses that colocalise with P2X7-R IR puncta. Co-localisation analysis indicates that P2X7-R IR puncta do not co-localise with ribbon synapses (E) but do show significant colocalisation with conventional synapses (F) in the IPL. Scale bars are 5 microns for A & C and 2 microns for B & D.

Although P2X7-R-IR puncta in the IPL co-localised with bipolar cell terminals it remained unclear whether this was direct expression of the receptor by the bipolar cells or amacrine cell expression at synapses in close association with the bipolar cell terminals. Further analysis of the expression profile of the P2X7-R-IR (red; Figure 9A–B, E) indicated that although the receptor was associated with VGLUT-IR bipolar cell terminals (blue) in the IPL, it was not co-localised with but likely post-synaptic to the bipolar specific, synaptic transmission ribbon protein, ribeye [41] (green). This suggests that the receptor is likely to be expressed by amacrine cells receiving input from bipolar cells (Figure 9A–B, E). In accordance with the concept that amacrine cells express the P2X7-R, the expression profile of the receptor at conventional synapses in the IPL was assessed using the marker Bassoon [42]. Many conventional synapses (Bassoon-IR; green) in the IPL were found to co-localise with P2X7-R-IR puncta (red) at sites distinct from the bipolar cell terminals (VGLUT, blue; Figure 9C–D, F).

**Figure 10:**
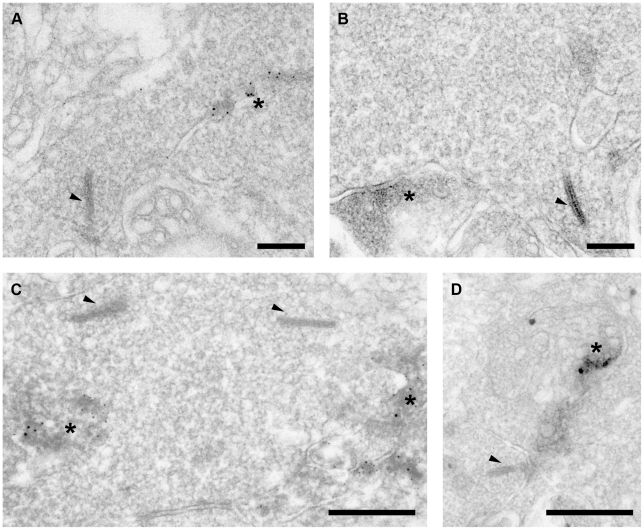
Transmission electron micrographs of the inner plexiform layer (IPL) of the WT mouse transverse retina following pre-embedding immunohistochemistry for P2X7-R-immunoreactivity. Ribbons are indicated by arrows and labelled amacine cells are indicated by astericks. (A), (B) & (C) P2X7-R immunoreactivity labels amacrine cells adjacent to rod bipolar cell ribbon synapses in the IPL. (D) A conventional amacrine cell synapse is labelled for P2X7 immunoreactivity in close proximity to a rod bipolar cell terminal. Scale bars are 200 nm for A & B and 500 nm for C & D.

To confirm these findings, the ultrastructural cellular location of the P2X7-R in the IPL was investigated using pre-embedding immunocytochemistry and electron microscopy (Figure 10). Putative amacrine cells that were post-synaptic to the rod bipolar cell ribbon synapse were labelled with the P2X7-R antibody, while the rod bipolar terminals were not (Figure 10A–C). In addition, amacrine cell conventional synapses also appeared to be labelled with the P2X7-R antibody (Figure 10D). These results are consistent with a role for the P2X7-R on amacrine cells, in modulating neuronal signalling in the IPL.

### The P2X7-R is expressed by astrocytes and microglia but not Müller cells

**Figure 11:**
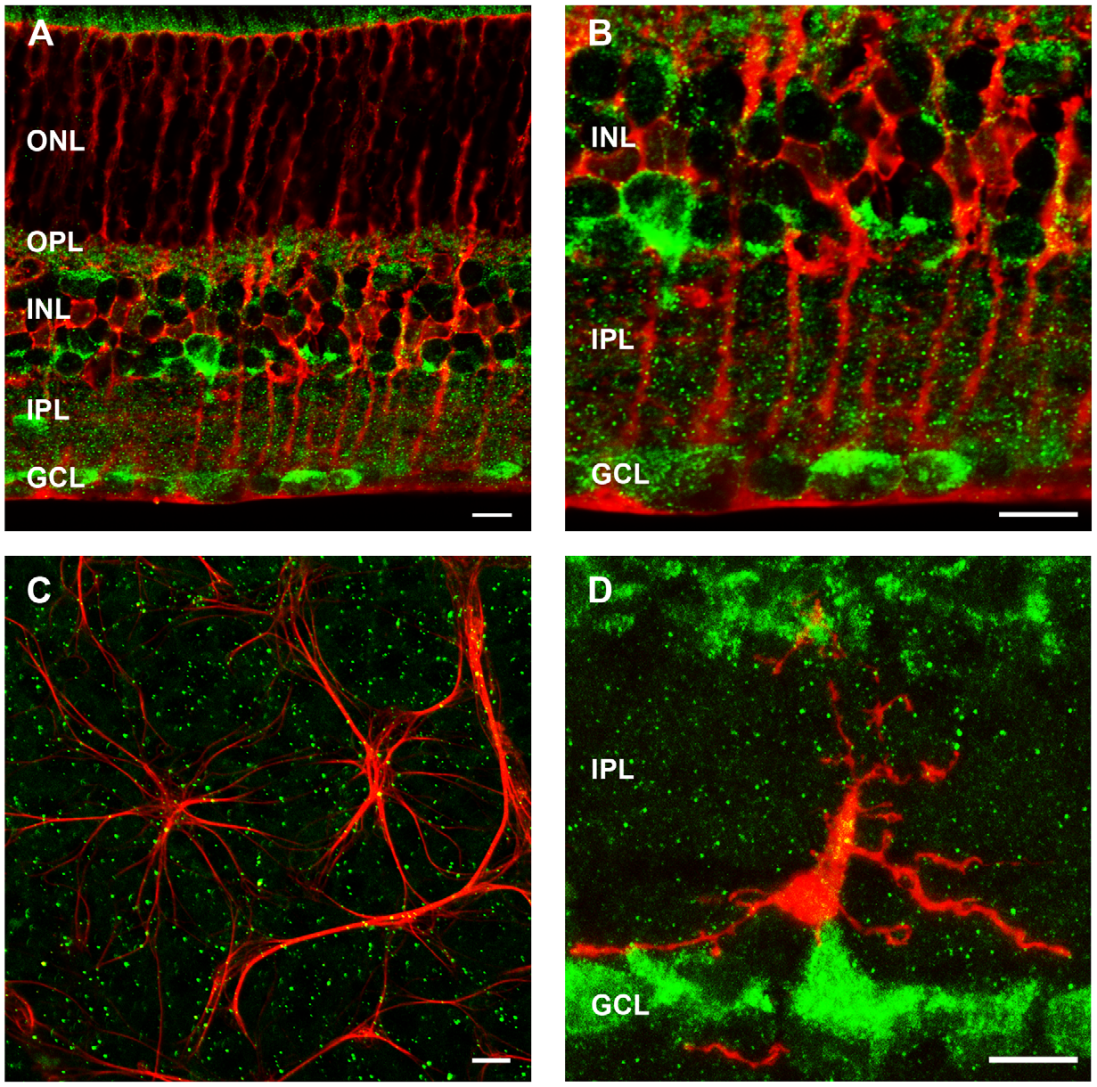
P2X7-R immunoreactivity (IR) is expressed by astrocytes and microglia but not Müller cells in the WT mouse retina. (A–B) Müller cells, labelled with an antibody against glutamine synthetise (red) were found to be closely associated with P2X7-R-IR puncta in the IPL (green), but not co-localised in the IPL. (C) Astrocytes, labelled with glial fibrilliary acidic protein (GFAP; red) and P2X7-R-IR (green) were found to co-localise in retinal flat mount. (D) Microglia labelled with mouse anti-GFP (pseudo-coloured red) from CX3CR1-GFP heterozygous mice were also found to co-localise with P2X7-R-IR (pseudo-coloured green). Scale bar, 10 microns.

Co-labelling for P2X7-R-IR and cell markers specific for macroglia and microglia was also undertaken. Müller cells, labelled with an antibody against glutamine synthetase (Figure 11A–B), were found to be closely associated with P2X7-R-IR puncta in the IPL but were not found to be significantly colocalised with the receptor above that expected by random chance (73611±15407 colocalised puncta/area of retina, mm^2^ observed vs 65762±18897 colocalised puncta/area of retina, mm^2^ random; n = 8, Student's t-test, p = 0.315). In contrast, astrocytes labelled with glial fibrilliary acidic protein (GFAP) in flat mount were found to co-localise with P2X7-R-IR puncta (4871±836 colocalised puncta/area of retina, mm^2^ observed vs 2492±547 colocalised puncta/area of retina, mm^2^ random; n = 8, Student's t-test, p = 0.004). In addition, microglia labelled with mouse anti-GFP (pseudo-coloured red) from CX3CR1-GFP heterozygous mice [43] were also found to co-localise with P2X7-R-IR (pseudo-coloured green, Figure 11D). This is consistent with studies in the brain and spinal cord that indicate the P2X7-R is important in regulating microglial activity [44].

## Discussion

In the current study we sought to determine if the P2X7-R has a role in mediating neurotransmission in the central nervous system, using the P2X7-KO mouse. Firstly we showed that the vesicular transporter for ATP, VNUT, which is required for ATP to act as a neurotransmitter at P2X7-Rs, is expressed by photoreceptors and also by cells of the inner retina in the mouse. Then, after showing that the P2X7-R (variant 1) is absent in the retina of P2X7-KO mice, we show that neuronal function is altered in the retina of these animals when compared with WT mice. The changes specifically in the inner retinal responses of the rod and cone pathways in the P2X7-KO mice are consistent with the immunocytochemical localisation of the receptor in the outer plexiform layer and on inner retinal neuronal pathways. These results imply that excitatory P2X7-R input to either photoreceptor terminals or inhibitory cells in the inner retina may modulate the rod and cone pathway responses in the normal retina.

### ATP neurotransmission in the retina

Vesicular storage and subsequent exocytosis of neurotransmitters is essential for chemical transmission in neuronal systems. Here we show that the vesicular transporter for ATP, VNUT is expressed by photoreceptors and also by cells of the inner retina in the mouse. This is consistent with other sensory systems, such as taste, where vesicular storage and release of ATP is a mode of signalling [45]. In the retina there is evidence that ATP may be co-released from amacrine cells (eg Cholinergic/GABAergic) [46], [47]. In addition extracellular ATP could be released from glia in the inner retina, such as astrocytes which have been shown to modulate neuronal signalling [11], [37]. Indeed hippocampal astrocyte cultures have been shown to express VNUT and also to store ATP in vesicles for exocytotic release [24], [48]. Although the exact cell classes that produce VNUT and store ATP as a neurotransmitter cannot be ascertained at this time, here we show that VNUT is expressed by photoreceptors and also cells of the inner retina indicating there is a mechanism for exocytotic release of ATP in the mouse retina.

Consistent with the notion that ATP transmission affects neuronal function the molecular machinery required for degradation of ATP has been shown near synapses that express purinergic receptors in the outer and inner retina [23]. Once released into the extracellular space, ATP is rapidly degraded by ectonucleotidases to ADP and/or AMP and then further degraded to adenosine [49]. In the retina, there is evidence for ectonucleotidase degradation of ATP at synaptic terminals [23] and both NTPDases1 and 2 have been found to be expressed immunocytochemically [50]. These findings support the hypothesis that ATP acts as a neurotransmitter, which may act at P2X7-Rs in the mouse retina.

### The P2X7-R variant 1 is absent in the P2X7-KO mouse

There are at least four mRNA splice variants of the P2X7-R. Variant 1 is the full length P2X7-R variant that generates a 595 amino acid peptide, including the critical C-terminal domain required for maximal ion channel function [25]. In the current study we show that while the mRNA and protein for the full length variant 1 of the P2X7-R is usually expressed in the retina of the WT mouse, the P2X7-KO ([14], Pfizer) mouse lacks this variant. This finding is expected given the P2X7-KO mouse has an insertion/deletion in the region of the receptor that encodes the C-terminal domain [14]. However, a previous study has shown that there may be read through of the P2X7-R variant 1 mRNA in the hippocampus of this P2X7-KO mouse such that a receptor with slightly altered electrophysiological properties is generated [26]. Our RT-PCR results indicate that there is no read through of the C-terminal mRNA of this receptor in the P2X7-KO mouse retina. In addition, our western blot results using an N-terminal specific antibody that would recognise all four variants of the receptor indicates that there is no full length variant 1 protein expressed in the retina or cortex of the P2X7-KO mouse. Our results are consistent with a recent study that shows the full length variant 1 of the P2X7-R is absent in the brain, salivary gland and spleen in the P2X7-KO ([14], Pfizer) mouse but that splice variants with a truncated C-terminus and significantly reduced function are still expressed [38].

### Neuronal function is altered in the P2X7-KO mouse

Neuronal function is altered in the mouse retina in the absence of the full length P2X7-R variant 1. Neuronal function was assessed in WT and P2X7-KO mice using the ERG. There was no change in rod photoreceptor function in the P2X7-KO mice suggesting that the P2X7-R does not modulate the photoreceptor outer segment response to light. In contrast the rod post-photoreceptor response (b-wave, PII) was significantly larger and faster in the P2X7-KO mouse and the amacrine cell derived OP3 response was also larger. Similarly the cone post-photoreceptor response was found to be enhanced in amplitude. This indicates that in the P2X7-KO mouse, rod and cone ON bipolar cells undergo larger depolarization in response to light than in the WT mouse. This could be due to an alteration in horizontal cell or amacrine cell feedback [51] or to a change in the response at the level of the photoreceptor terminal, which can have altered glutamate release even in the presence of normal outer segment hyperpolarisation to light [52]. Consistent with the current finding of enhanced inner retinal responses in the absence of the P2X7-R, administration of a P2X-R agonist (Bz-ATP) with 10–30 fold selectivity for the P2X7-R has been found to reduce the post-photoreceptor responses (b-wave, PII) of both the rod and the cone pathways in the rat [23]. These data suggest that under normal conditions, activation of P2X7-Rs may enhance glutamate release at the level of the photoreceptor terminal or enhance inhibitory output from horizontal or amacrine cells onto rod and cone ON bipolar cells thereby reducing the amplitude of their depolarising response to light. This effect may be important in modulating the timing of the ON pathway response or in modulating the encoding of the spatial properties of the environment. The idea that the P2X7-R may play a role in spatial resolution is supported by the finding that the P2X7-KO mice displayed slightly enhanced photopic visual acuity compared with their WT counterparts.

### The P2X7-R is expressed by neurons of the inner retina

Both P2X7-R mRNA and protein have been detected in neuronal cells within the rat and primate retina previously [16], [17], [23], [53], [54]. Here we show in the mouse that the P2X7-R is expressed in the outer plexiform (OPL), inner nuclear (INL), inner plexiform (IPL) and ganglion cell (GCL) layers. Specifically, in the OPL it is expressed within PSD-95 labelled photoreceptor terminals where it is either expressed by horizontal cells processes as indicated in figure 8A and/or by the photoreceptor terminals themselves [17]. In the inner retina it colocalises with rod bipolar cells, type 2 and 6 cone bipolar cells, inhibitory GABAergic amacrine cells and also ganglion cells. [17]

With further analysis, we found that the receptor is expressed, not by bipolar cells, but by amacrine cell synapses that are post-synaptic to rod bipolar cell terminals and in close association with the bipolar cell ribbon synapses. In addition, the receptor was found to colocalise with conventional synapses in the inner plexiform layer suggesting involvement in amacrine-amacrine and amacrine-ganglion cell synaptic transmission. This suggests that in the inner retina the receptor is involved in providing excitatory input to amacrine cells that provide feedback to the rod bipolar cells and also in excitatory signalling between amacrine and ganglion cells.

These P2X7-R immunolocalisation findings in the mouse retina are similar to those from a previous study from our laboratory completed in the rat retina using a C-terminal directed antibody [17]. In the rat retina, P2X7-R-IR was found on photoreceptors at the ultrastructural level and within PSD-95 labelled photoreceptor terminals, as well as on horizontal cells, amacrine cells and also ganglion cells [17]. In the current study we did not use the C-terminal directed antibodies, which should only label Variant 1 of the P2X7-R, as these were found to label similarly in the WT and P2X7-KO. However, the correlation in the neuronal labelling profile of P2X7-R-IR in the rat, using the C-terminal directed antibody [17], and here in the mouse, using the N-terminal directed antibody, suggests that both antibodies may indeed label P2X7-R protein. This suggests that the C-terminal directed antibody may be labelling other P2X7-R splice variants, a receptor associated protein of very low or high molecular weight that was not picked up on the western blot as has been seen to occur with glycine receptor antibodies [55], [56], or an as yet uncharacterised retinal specific, C-terminal splice variant of the P2X7-R that is still expressed in the P2X7-KO mouse. Therefore, both the C-terminal and N-terminal directed P2X7-R antibodies may be of use in determining expression of P2X7-R in the mouse and rat retina.

In addition to identification of the neuronal location of the P2X7-R in the retina, further analysis of microglial and macroglial expression was undertaken. P2X7-R-IR was found on microglia, the resident macrophages of the central nervous system. This is consistent with a myriad of studies in brain and spinal cord that suggest the receptor is important in mediating a neuro-inflammatory response in disease ([44], for review). Müller cells along with astrocytes represent the macroglia of the retina. There is currently debate in the literature as to whether Müller cells express the P2X7-R. It has been suggested that Müller cells do not express P2X7-R mRNA [57] or protein [16]. However, other studies suggest that they do produce P2X7-R mRNA and protein, and these studies provide additional functional evidence for P2X7-R mediated calcium currents in Müller cells induced by BzATP [21]. However, in the present study, although P2X7-R-IR was closely associated with Müller cell processes, it did not co-localise, while in contrast, P2X7-R-IR did co-localise with astrocytes. Thus further research is required to determine the role of the P2X7-R in modulating macroglial function in the retina.

### The role of the P2X7-R in the normal retina

The functional data, together with the immunocytochemical analysis, imply that the P2X7-R is usually expressed on neurons, microglia and astrocytes in the retina and that it is involved in regulating the inner retinal responses of the rod and cone pathways. This regulation could be at the level of the photoreceptor terminal or at the level of horizontal or amacrine cell feedback. The location of the receptor on amacrine cells in close association with the rod bipolar cell ribbon synapse suggests that activation of P2X7-R on amacrine cells may induce greater release of eg GABA to provide inhibition of rod bipolar cell output. In the normal retina, the P2X7-R likely provides excitatory input to either photoreceptor terminals or to inhibitory cells that shape both the rod and cone pathway response.
